# Malpractice claimed calls within the Swedish Healthcare Direct: a descriptive – comparative case study

**DOI:** 10.1186/s12912-021-00540-3

**Published:** 2021-01-14

**Authors:** Annica Björkman, Maria Engström, Ulrika Winblad, Inger K. Holmström

**Affiliations:** 1 Faculty of Health and Occupational Studies, University of Gavle, Gävle, Sweden; 2 Department of Public Health and Caring Sciences, Uppsala University, Uppsala, Sweden; 3 School of Health, Care and Social Welfare, Mälardalen University, Västerås, Sweden

**Keywords:** Telephone advice nursing, Patient safety, Medical errors

## Abstract

**Background:**

Medical errors are reported as a malpractice claim, and it is of uttermost importance to learn from the errors to enhance patient safety. The Swedish national telephone helpline SHD is staffed by registered nurses; its aim is to provide qualified healthcare advice for all residents of Sweden; it handles normally about 5 million calls annually. The ongoing Covid-19 pandemic have increased call volume with approximate 30%. The aim of the present study was twofold: to describe all malpractice claims and healthcare providers’ reported measures regarding calls to Swedish Healthcare Direct (SHD) during the period January 2011–December 2018 and to compare these findings with results from a previous study covering the period January 2003–December 2010.

**Methods:**

The study used a descriptive, retrospective and comparative design. A total sample of all reported malpractice claims regarding calls to SHD (*n* = 35) made during the period 2011–2018 was retrieved. Data were analysed and compared with all reported medical errors during the period 2003–2010 (*n* = 33).

**Results:**

Telephone nurses’ failure to follow the computerized decision support system (CDSS) (*n* = 18) was identified as the main reason for error during the period 2011–2018, while failure to listen to the caller (*n* = 12) was the main reason during the period 2003–2010. Staff education (*n* = 21) and listening to one’s own calls (*n* = 16) were the most common measures taken within the organization during the period 2011–2018, compared to discussion in work groups (*n* = 13) during the period 2003–2010.

**Conclusion:**

The proportion of malpractice claims in relation to all patient contacts to SHD is still very low; it seems that only the most severe patient injuries are reported. The fact that telephone nurses’ failure to follow the CDSS is the most common reason for error is notable, as SHD and healthcare organizations stress the importance of using the CDSS to enhance patient safety. The healthcare organizations seem to have adopted a more systematic approach to handling malpractice claims regarding calls, e.g., allowing telephone nurses to listen to their own calls instead of having discussions in work groups in response to events. This enables nurses to understand the latent factors contributing to error and provides a learning opportunity.

## Background

Medical errors are reported as a malpractice claim, and it is mandatory in Sweden for healthcare providers to report risk of medical errors and events that have led to or could have led to a medical error to the responsible authority [1]. Healthcare providers are responsible for the investigation, e.g., for identifying factors contributing to the medical error and for facilitating learning from the medical error [2]. Due to limited healthcare resources and politicians’ demands for cost reduction, nurse-led telephone advice nursing (here referred to as “telephone nursing”) is rapidly increasing. In Sweden, the national telephone nursing service Swedish Healthcare Direct (SHD) is recommended as the populations’ first contact with the healthcare system [3, 4]. The aim of telephone nursing services is to provide increased accessibility to qualified healthcare advice and to rationalize use of limited healthcare resources [5, 6]. Telephone nursing includes triage of care-seekers’ need for care, referral to the appropriate level of care, offering self-care advice and supporting care-seekers [3, 7]. The telephone nurses independently triage care-seekers’ need for care using the mandatory assistance of a computerized decision support system (CDSS) [8, 9]. The CDSS is symptom based, and the telephone nurses enter the CDSS using as a search term the main symptom presented by the caller. Despite use of a CDSS to increase patient safety, medical errors do occur within telephone nursing [10], and these errors are reported as malpractice claims [2]. A medical error can be defined as “*the failure of a planned action to be completed as intended or the use of a wrong plan to achieve an aim”* [11]. The new Patient Safety Law from 2011 [1] placed the responsibility for investigating the medical error and taking the measures needed to prevent/protect patients from further medical errors on the healthcare provider. The law was later revised [2] by prescribing the measures healthcare providers should take when an event resulted or could have resulted in severe medical error for the patient involved. It also stressed the importance of *learning* from medical errors; knowledge transfer is reported to be of the utmost importance to successful patient safety work. When the preceding law was in force [12], it was the responsibility of the Swedish Board of Health and Welfare to conduct an investigation (root cause analysis) to identify what went wrong, and why, when a patient was affected by a medical error. In a previous study [10], i.e. when the preceding law was in force, we investigated all malpractice claims and healthcare providers’ measures following telephone calls to SHD. In order to describe and understand how the new law [1, 2] has influenced patient safety work within telephone nursing, we collected new data for the period 2011–2018.

In Sweden, the telephone nursing service SHD is organized as a network to which all the regions are connected; each region is responsible for its own call centre. There are 33 call centres across the country. The service is reached through a national telephone number (1177). All SHD sites have the same structure; hence, the telephone nurses work in a call centre without physical contact with care-seekers. The telephone nurses are obliged to use an CDSS developed in-house. This includes medical information on symptoms, guidelines and questions as well as documentation in the patient records. The CDSS used is accessed by entering the main symptom presented by the care-seeker, and problems arise when callers present a range of complex problems [8, 13, 14]. Hence, the CDSS constrains the telephone nurses to choosing one main symptom, and nurses tend to pose questions that request confirmation of the absence rather than the presence of symptoms [15]. The information and guidelines are assessed by medical expertise regularly to assure high quality and up-to-date information. However, interview studies have shown that telephone nurses do not always use the CDSS as intended [14] and the image of safety may be compromised.

Telephone nursing is complex; telephone nurses rely on communicative skills to gain the information they need as the basis for their assessments [3, 16]. The process of telephone nursing was described by Greenberg as dynamic and goal-oriented, where telephone nurses work in three phases: gathering information, cognitive processing, and output [16]. However, telephone nurses’ communication with callers seems to be affected by CDSS use, as more closed-ended questions are asked and the dialogue focuses mainly on symptoms, which entails the risk that other relevant aspects will be ignored [17]. Such aspects might be pivotal, as Gamst-Jensen et al. [18] showed the importance of exploring callers’ concerns so as to acquire more contextual information and, hence, a richer picture of the situation.

One systematic review suggested that using CDSS to support clinical decisions improves patient care significantly [19]. On the other hand, another systematic review [20] revealed that implementation of CDSS does not always have a positive outcome and that use of the tool requires further evaluation. Patient safety in telephone nursing can be enhanced by using a CDSS [7, 8], but other aspects may affect the triage process. The gender of the care-seeker [3], callers self-rated worry [21] might affect the telephone nurses, and cues of physical dominance (voices with a low fundamental frequency and formant frequencies) have been shown to lead to higher evaluation of medical emergency [22]. In addition, limiting the time for each call, to increase accessibility, can result in stress [23] and, thus, negatively affect patient safety. These finding add to the questions surrounding patient safety within telephone nursing. In a previous study [10], we analysed the characteristics of all malpractice claims arising from calls to SHD during the period 2003–2010 (*n* = 33). Since the latter study, the number of calls to SHD has increased and, today, SHD is one of Sweden’s largest healthcare providers. The present study was done before the Covid-19 pandemic. In Sweden, where the study was conducted, there have been no substantive changes in the delivery of the national telephone nursing advice service due to the pandemic, though the volume of calls to the service have increased by over 30%. In our opinion, the increase in call volume, highlights the relevance of the study. For this reason, studies on patient safety work within SHD are of importance.

Malpractice reporting is an important measure in patient safety work. As mentioned, the malpractice reporting system in Sweden is a mandatory no-fault system that differs substantially from the tort litigation systems used in the United States, which compensate patients financially if something goes wrong [24]. In Sweden, approximately 1400 patients die annually and 110,000 patients are affected by a medical error [25]. However, Anderson and Abrahamson [26] showed that less than 10% of medical errors are reported in Sweden. The healthcare sector in Sweden has become increasingly financially restrained, with consequences for the working environment and high turnover rates among registered nurses (RNs). Simultaneously, technical development has enhanced the telephone system, CDSS and information technology used by telephone nurses. All of these factors have the potential to affect the number of medical errors in telephone nursing either positively or negatively, which is why we wished to conduct this follow-up of our previous study.

## Method

### Aim

The aim of the present study was twofold: to describe all malpractice claims and healthcare providers’ reported measures regarding calls to Swedish Healthcare Direct (SHD) during the period January 2011–December 2018 and to compare these findings with results from a previous study covering the period 2003–2010 [10].

Specific research questions for the study were:

1. What were the characteristics of malpractice claims for calls made during the period 2011–2018 compared to 2003–2010?
2. What were the identified causes of medical errors during the period 2011–2018 compared to 2003–2010?
3. What were the reported patient-related consequences of the malpractice claims for calls made during the period 2011–2018 compared to 2003–2010?
4. What were the healthcare providers’ reported measures during the period 2011–2018 compared to 2003–2010?

### Design

The study used a descriptive, retrospective and comparative design.

### Data source and material

When a patient is affected by a medical error in Sweden, is it mandatory for healthcare providers to submit a report to the authority responsible for supervision and control of the Swedish healthcare system and social welfare. It is the healthcare provider’s responsibility to both report and investigate the event. The investigation should map the event, identify contributing factors and provide suggestions regarding measures to prevent the error from reoccurring. It is the authority’s responsibility, however, to ensure that these events have been properly investigated and that the measures taken by the healthcare provider are sufficient. Furthermore, it is the authority’s responsibility to share information on the reported events with other healthcare providers [1, 2]. The data for the present study consist of such investigations of reported malpractice claims within SHD as well as the organization’s response to malpractice claims for calls made to SHD during the period January 2011–December 2018. The data is collected over a long period of time, the first data collection was done January 2003 until end of December 2010 and the data for the follow-up was collected from January 2011 until end of December 2018, using a retrospective design. All malpractice claims (*n* = 35) regarding SHD during the period 2011–2018 were retrieved as text documents via the local (*n* = 7) registrars for the responsible authority during February–July 2019. At the time of the study, all of Sweden’s councils were connected to SHD. The investigations varied in length from four to 12 pages and did not use standardized categories for causes of medical errors. One root-cause analysis could describe more than one reason for the medical error (see Table 4), and the organizations’ response could consist of more than one measure (see Table 5).

### Data analysis

The content of the text files was analysed using summative content analysis [27]. Text describing the care-seeker’s reason for calling, the IVO’s description of what went wrong and the organization’s response to the malpractice claims were condensed without changing their content and grouped into categories and sub-categories. This categorization was conducted by author AB, with author ME acting as co-coder in seven cases. In the analysis of the categories, each case’s reported causes or measures were dichotomized to 0) no causes or measures were reported in the category and 1) one or more causes were reported for the case in the category. Descriptive and comparative (Fisher’s exact test) statistics were used to compare categories for 2003–2010 with those for 2011–2018.

## Results

The analysis showed that in 17 out of 35 (48.6%) cases during the period 2011–2018, there was more than one call to SHD in connection with the malpractice claim; see Table 1. Corresponding figures for 2003–2010 were 14 out of 33 cases (42%). During the period 2011–2018, 26 of the calls had been made by the patients themselves, nine by a relative (a guardian/parent, eight female and one male guardian/parent). In 16 calls, there was no information on who made the call to SHD. Similar results were found for 2003–2010, as 25 calls had been made by the patients themselves; ten of the calls regarding adults had been made by a relative or by a friend (eight calls regarding children had been made by mothers and two by fathers). These findings indicate that mothers typically contact SHD.

**Table 1:** Characteristic of cases and calls

	2011–2018		2003–2010	
Gender, male/female	14/16 5 unknown		19/14	
Age, median/mean (range)	39/45 (10 months to 86 years) 17 cases no info. on age, 2 mentioned as children.		41/48 (1 to 80 years)	
Number of calls in each case (i.e., some cases made more than one call)	1 call	18 cases	1 call	11 cases
	2 calls	11 cases	2 calls	10 cases
	3 calls	4 cases	3 calls	4 cases
	4 calls	2 cases		
Total cases/calls	35/60		33/45	

Female patients (*n* = 16/35) were in the majority of the malpractice claims for calls made during the period 2011–2018, and male patients were in the majority for 2003–2010 (*n* = 19/33). However, in five of the documents, gender had been blinded by the authority, and gender aspects should therefore be treated with caution. Median age values for the patients were 39 years (2011–2018) and 41 years (2003–2010).

Like in our previous study, the severity of patient injury in the reported events is high. In the present study, 10/35 (29%) of the affected patients died, and for the period 2003–2010, 13/33 (39%) patients died; see Table 2.

**Table 2:** Description of the consequences for affected patients in the malpractice claims

What happened to patients? n	2011–2018, *n* = 35	2003–2010, *n* = 33
Death	10	13
Admitted to ICU/MICU	11	12
Admitted to standard care	12	7
Leave hospital after treatment		1
No information provided	2	

*ICU* Intensive care unit, *MICU* medical intensive care unit

### Reason for calling SHD

In the malpractice claims, fever was the most common reason for calling SHD (*n* = 6) during the period 2011–2018 and abdominal pain (*n* = 11) was the most common reason for 2003–2010. Abdominal pain was the second most common reason for 2011–2018 and chest pain for 2003–2010. Chest pain was the third most common reason for 2011–2018 and fever for 2003–2010. See Table 3 for further description of reasons for calling SHD.

**Table 3:** Reason for calling SHD

Reason for calling	2011–2018, ***n*** = 35	2003–2010, ***n*** = 33
Fever	6	3
Abdominal pain	5	11
Chest pain	4	6
Bodily Pain	3	
Breathing problems	3	3
Gastroenteritis	3	
Neurological symptoms	3	
Rash	3	
Trauma	2	2
Abnormal urination	1	
Eye problems	1	
Poor general condition	1	2
Animal bite		1
Cold, flu		1
Dizziness		3
Headache		1

### The identified causes of the medical error

The three most common reasons (categories) for the error during the period 2011–2018 were compared with the findings for 2003–2010. The results revealed that errors related to telephone nurses’ communication were less frequent for 2011–2018 compared to 2003–2010 (*p* = 0.028). For the other two categories – decision process and organizational deficits – the results were non-significant when comparing the two datasets; see Table 4.

**Table 4:** The central authority’s description of cause of malpractice claim (why)

Category^**1**^	Sub-category	2011–2018	2003–2010
**Communication, n** 14 (2011–2018) 22 (2003–2010) *p* = 0.028^1^	Inadequate anamnesis (too few questions)	7	10
	Communication failure	6	11
	Failure to listen to caller	1	12
	Talked through third party	4	1
	Did not follow up on caller’s understanding		1
**Decision process** 27 (2011–2018) 21 (2003–2010) *p* = 0.222^1^	Probability diagnosis	7	8
	Did not follow/use CDSS	18	7
	Lack of overall picture of caller	1	5
	Did not follow guidelines	7	6
	Did not reconsider previous diagnosis	3	3
	Deficit in documentation of call	2	
**Organizational deficits** 10 (2011–2018) 17 (2003–2010) *p* = 0.053^1^	Lack of personal competence	1	9
	High workload	6	6
	Long work shift (> 9 h)	1	
	Deficit in CDSS	3	5
	Work task not defined		3
	Lack of healthcare resources		1
	Lack of support	1	

^1^ In the analysis of the categories, each case’s reported causes or measures were dichotomized to 0) no causes or measures were reported in the sub-category and 1) one or more causes were reported for the case in the sub-category

### Healthcare providers’ measures

Measures targeting the individual nurse were reported in 16 cases for the period 2011–2018 and no cases for 2003–2010. Listening to one’s own calls as a measure taken by the organization was reported in 16 cases for 2011–2018, and not at all for 2003–2010. Staff education (*n* = 21) was the most common measure taken within the organization during the period 2011–2018; for 2003–2010, the most common measure was discussion in work group (*n* = 13). As in our previous study (ref), several measures could have been taken for each case; see Table 5.

**Table 5:** The measures taken by the organization (SHD) in response to malpractice claims (sometimes several measures were taken for each case)

Category^1^	Sub-category	2011–2018	2003–2010
**Measure targeting staff (group level), n** 23 (2011–2018) 21(2003–2010) *p* = 0.858^1^	Discussion in work group	9	13
	Education of staff	21	10
**Measure targeting the organization** 1 (2011–2018) 0 (2003–2010)	Collaboration with other units within SHD	1	0
	Increase of staffing	1	0
**Measure targeting the structure/guidelines** 5 (2011–2018) 13(2003–2010) *p* = 0.019^1^	Revision of guidelines	3	8
	Revision to CDSS	5	6
**Measure targeting the individual (the nurse)** 16 (2011–2018) 0 (2003–2010)	Listening to own calls/coaching sessions	16	0
	Psychological support to affected staff	1	0
**No measure reported** 7(2011–2018) 7 (2003–2010) *p* = 0.902	No measure reported	7	4
	Measures planned, not specified		3

^1^ In the analysis of the categories, each case’s reported causes or measures were dichotomized to 0) no causes or measures were reported in the sub-category and 1) one or more causes were reported for the case in the sub-category

## Discussion

Like in our previous study [10], the severity of the reported malpractice cases, e.g. the severity of patient injury, is high. A possible explanation, and limitation, of the study is that only the most severe cases are reported to the authority (see 10). In the present study, ten out of 36 (28%) of the affected patients died as a result of the error, compared to 13 out of 33 (39%) for the period 2003–2010. The investigations performed by the healthcare providers showed that, during the period 2011–2018, the medical errors were most commonly caused by telephone nurses’ failure to follow or use CDSS (*n* = 18). This is an interesting finding, as the telephone nurses employed at SHD are obliged to use the CDSS to guide their assessments and carry out the mandatory documentation. Previous studies have shown that telephone nurses do not use the CDSS as intended [14, 15], for instance by using it to confirm decisions they had already made, e.g. nurses learn to manipulate the CDSS algorithms to ensure that the CDSS outcome is in line with their own ideas about what is right for the patient. One possible limitation of the study is that investigations are made by many different healthcare providers, and their knowledge preforming such investigations may vary. Observational studies [14] have revealed how experienced telephone nurses use the CDSS after the calls, only to confirm their assessments. Other studies [28] have also concluded that telephone nurses’ usage of the CDSS seems to be guided by their own experience and ability to adapt the CDSS to align with local clinical practice [29]. This inconsistency between CDSS recommendations and clinical practice routines and guidelines creates problems for telephone nurses, as the healthcare providers often report that telephone nurses over-triage care-seekers’ need for care, creating a tension between SHD and healthcare providers [30]. Two systematic reviews [31, 32] have concluded that there are problems associated with the appropriateness of telephone advice nursing, and under-referral and under-estimation of urgency were found.

Regarding errors related to telephone nurse’s communication, these were less frequent during the period 2011–2018 (*n* = 18) compared to 2003–2010 (*n* = 35) (*p* = 0.0281). This could be a result of the measure listening to one’s own calls/coaching sessions (*n* = 16), which had been introduced in the organizations in response to the medical error. However, medical errors caused by communication failure and telephone nurses asking too few questions were still common. Communication is at the core of the telephone nursing process [16], and as in most healthcare communication, there is a power differential between the different actors [33]. When telephone nurses reflect over what contributes to malpractice claims, they report that they often expect callers to make the final decision regarding appropriate measures. Situations leading to a malpractice claim were described, one of which is when callers were advised to contact emergency services if they felt their condition had worsened, but did not follow this advice [34]. This strategy could be interpret as “passing the buck”, which is not at all appreciated by the caller [35]. The callers reported feeling that the telephone nurses used “the safe approach”, e.g., leaving the caller to decide whether to wait and see or to seek medical assistance. Improvement of communication between healthcare professionals and patients is essential to successful patient safety work [36] and patient concordance. In studies investigating the actual communication between telephone nurses and callers, the results show that telephone nurses’ communications seemed to be nurse-driven, with few open-ended questions and a lack of exploration of callers’ understanding of the advice given [10, 37]. The communication found in these studies is not in line with the Dialogue Process, and it is as yet unknown to what extent telephone nurses employed within SHD adhere to the process they have been taught. Listening to one’s own calls in connection with a medical error is one way to develop communication skills, and effective communication skills are considered a key competence for telephone nurses. Despite the important role of telephone nurses, there is a lack of standardized education in telephone nursing [38].

In our previous study, the investigations showed that organizational factors such as high workload (*n* = 6) contributed to the medical error reported in the malpractice claims, but measures targeting the organization were sparse (*n* = 1). Several studies performed within the context of telephone nursing have pointed out how telephone nursing is perceived as stressful work [29, 30]. In an interview study [34], the telephone nurses who had been exposed to a malpractice claim revealed how always being aware of the number of calls waiting, and always feeling the pressure of organizational goals, could result in premature termination of calls. Other aspects of the telephone nurses’ work environment may pose a threat to patient safety. As shown [29, 39], disturbing background sound caused by callers [39] and disturbing sounds from other colleagues due to insufficient workplace soundproofing [29] might disturb the communication. Within the context of nursing, work environment has also shown to be of importance to the outcome of care, e.g. patient safety [40], and increased patient mortality [41].

Despite the introduction of a new Patient Safety Act [1, 2] it seems that not a great deal has changed regarding the organization’s measures as a response to the malpractice claim, but some tendencies can be seen. The present study shows that healthcare providers’ response to the malpractice claims entails new measures targeting the individual nurse, e.g. measures such as listening to one’s own calls and coaching sessions in response to the medical error. However, this indicates that the organization’s measures still focus mainly on the individual’s active failure than on the underlying latent factors [42, 43]. In our previous study [10], the measures taken by the organizations in response to the malpractice claims focused on discussion in work groups and staff education, e.g., individual active failure [42]. Active failure is defined as unsafe acts performed by individuals in direct contact with the patient. Latent failure, e.g., inevitable “resistant pathogens”, is defined as the stressful work environment, under-staffing and inexperience found within the organization – the system. It is reasonable [42, 44] to also argue that active failures often occur due to insufficient support from the latent conditions. Findings from both of our studies [10] show that, despite the factors contributing to the medical errors that derive from organizational deficits (*n* = 10 for 2011–2018; *n* = 17 for 2003–2010), organizations’ measures mainly target staff (*n* = 23 for 2011–2018; *n* = 21 for 2003–2010). The investigations performed by the responsible authority identify active failures made by individuals, such as telephone nurses’ failure to use the CDSS and use of a probability diagnosis as the cause, but they do not investigate WHY these active failures occur. When the focus is on active failures the errors are likely to reoccur, because the true causes – found within the organization (latent conditions) – not have been identified and addressed [42, 44]. As shown in the present study and several previous studies, telephone nurses’ work environment and organizational factors, such as high-work load and limited possibilities for learning, need to be addressed. Hence, there needs to be a balance between what is expected of telephone nurses and their ability to carry out their work, e.g., which are prerequisites to providing high-quality, patient-safe care.

## Conclusion

The proportion of malpractice claims in relation to all patient contacts to SHD is still very low; it seems that only the most severe patient injuries are reported. The fact that telephone nurses’ failure to follow the CDSS is the most common reason for error is notable, as SHD and healthcare organizations stress the importance of using the CDSS to enhance patient safety. The healthcare organizations seem to have adopted a more systematic approach to handling malpractice claims regarding calls, e.g., allowing telephone nurses to listen to their own calls instead of having discussions in work groups in response to events. This enables nurses to understand the latent factors contributing to error and provides a learning opportunity.

## Data Availability

The data that support the findings of this study are available from the Swedish healthcare system and social welfares local registrars but restrictions may apply to the availability of these data, which were used under license for the current study, and so are not publicly available. Data are however available from the authors upon reasonable request and with permission of the Swedish healthcare system and social welfares local registrars.

## Abbreviations

CDSS: Computerized decision support system
SHD: Swedish healthcare Direct
